# Matrine promotes mitochondrial biosynthesis and reduces oxidative stress in experimental optic neuritis

**DOI:** 10.3389/fphar.2022.936632

**Published:** 2022-09-27

**Authors:** Yifan Song, Mengru Wang, Suyan Zhao, Yanjie Tian, Chun Zhang

**Affiliations:** ^1^ Department of Ophthalmology, Peking University Third Hospital, Beijing, China; ^2^ Beijing Key Laboratory of Restoration of Damaged Ocular Nerve, Peking University Third Hospital, Beijing, China; ^3^ Department of Pharmacology, School of Basic Medical Sciences, Zhengzhou University, Zhengzhou, China

**Keywords:** matrine, optic neuritis, mitochondrial biosynthesis, oxidative stress, SIRT1, PGC-1α, Nrf2

## Abstract

Optic neuritis (ON), characterized by inflammation of the optic nerve and apoptosis of retinal ganglion cells (RGCs), is one of the leading causes of blindness in patients. Given that RGC, as an energy-intensive cell, is vulnerable to mitochondrial dysfunction, improving mitochondrial function and reducing oxidative stress could protect these cells. Matrine (MAT), an alkaloid derived from *Sophora* *flavescens*, has been shown to regulate immunity and protect neurons in experimental autoimmune encephalomyelitis (EAE), an animal model of multiple sclerosis and ON. However, the protective mechanism of MAT on RGCs is largely unknown. In this study, we show that MAT treatment significantly reduced the degree of inflammatory infiltration and demyelination of the optic nerve and increased the survival rate of RGCs. The expression of Sirtuin 1 (SIRT1), a member of an evolutionarily conserved gene family (sirtuins), was upregulated, as well as its downstream molecules Nrf2 and PGC-1α. The percentage of TOMM20-positive cells was also increased remarkably in RGCs after MAT treatment. Thus, our results indicate that MAT protects RGCs from apoptosis, at least in part, by activating SIRT1 to regulate PGC-1α and Nrf2, which, together, promote mitochondrial biosynthesis and reduce the oxidative stress of RGCs.

## Introduction

Optic neuritis (ON), a common cause of visual defects in young people, is an inflammatory disease of the optic nerve, which is characterized by immune cell infiltration and subsequent demyelination (Kobayter and Chetty, 2019; Pihl-Jensen et al., 2021). Patients typically experience a sharp decrease in vision, orbital pain aggravated by eye movements, an afferent papillary defect, and dyschromatopsia, with or without swelling of the optic nerve head. ON is the initial manifestation in approximately 20% of multiple sclerosis (MS) patients, and 50% of ON patients will develop MS during the disease (Kimura et al., 2017). Severe, even irreversible, visual loss can occur in ON, neuromyelitis optica (NMO), and experimental autoimmune encephalomyelitis (EAE), an animal model of MS (Azuchi et al., 2017).

There is increasing evidence that irreversible vision loss may result from inflammation of the optic nerve and apoptosis of the retinal ganglion cells (RGCs) (Liu et al., 2017; Zhang et al., 2020). Because the long axons of RGCs are vulnerable to lack of energy, they are highly reliant on mitochondria and are sensitive to mitochondrial dysfunction and oxidative stress (Lin and Kuang, 2014). Indeed, mitochondrial oxidative stress can induce neuronal damage in experimental ON (EON) (Qi et al., 2007). The activation of sirtuin 1 (SIRT1), a member of an evolutionarily conserved gene family (sirtuins) encoding NAD+ dependent deacetylases that regulates various components of cellular metabolism (Martin et al., 2015), can prevent RGC loss in ON by reducing oxidative stress and promoting mitochondrial function in neuronal cell lines (Khan et al., 2012). SIRT1 may function *via* regulating the expression of peroxisome proliferator-activated receptor γ coactivator-1α (PGC-1α), a metabolic coactivator that induces mitochondrial biogenesis and respiration by interacting with transcription factors (Finck and Kelly, 2006; Gerhart-Hines et al., 2007). SIRT1 also plays an important role in regulating the expression and activation of nuclear factor erythroid 2-related factor 2 (Nrf2) (Ding et al., 2016), a basic leucine zipper transcription factor that activates the gene network related to antioxidant defense and cell detoxification (Johnson and Johnson, 2015).

Matrine (MAT), a natural alkaloid component extracted from the herb Radix Sophorae Flavescentis, with a molecular weight (MW) of 258.43 (C15H24N2O), has been widely used in the clinical treatment of human hepatitis B and leukocytopenia with very few adverse reactions (Wang et al., 2017). Previous studies have shown that MAT can significantly improve the neural function of EAE and reduce central nervous system (CNS) and peripheral inflammatory responses (Kan et al., 2017; Zhang et al., 2017). Recent studies have shown that MAT can enhance mitochondrial function and inhibit oxidative stress in oligodendrocytes of EAE (Wang et al., 2019). In addition, we have conducted a preliminary exploration of the protective effect of MAT in RGCs in EON (Kang et al., 2021); however, the mechanism underlying this effect has not yet been elucidated. We focused on this issue in the present study and found that MAT regulates the expression and activation of PGC-1α and Nrf2 by activating SIRT1, promotes mitochondrial biosynthesis, and reduces oxidative stress, thus having a therapeutic effect on EON.

## Materials and methods

### Animals and EAE induction

Female Wistar rats (6–8 weeks old, 180–200 g) were purchased from the Jinan Pengyue Experimental Animal Breeding Co., Ltd., China, and raised under the condition of specific pathogens at the Laboratory Animal Center of the Henan Academy of Chinese Medicine, China. EAE was induced as described previously (Chu et al., 2021). Briefly, the spinal cord homogenate of guinea pigs (Jinan Jinfeng Experimental Animal Co., Ltd., China) was emulsified with the same volume of complete Freund’s adjuvant (Sigma, St. Louis, MO, United States) containing 6 mg/ml Bacillus Calmette–Guérin vaccine (Solarbio Bio-Technology Co., Shanghai, China). Each rat was injected subcutaneously at four separate sites on the back with 0.5 ml of antigen emulsion.

## Ethics approval and consent to participate

This study was approved by the Ethics Committee of Scientific Research of Henan Academy of Chinese Medicine; the ethical review number is HNTCMDW-20170601. All of the protocols were approved, and every effort was made to ensure minimal animal suffering.

### MAT treatment and clinical scoring

The immunized rats were stochastically divided into three groups (*n* = 10, each group) (Kobayter and Chetty, 2019): EAE rats were injected intraperitoneally (i.p.) with 250 mg/kg/day MAT (Meilunbio, Dalian, China) (Kan et al., 2013) (Jiangsu Chia Tai-Tianqing Pharmaceutical Co. Ltd., Jiangsu, China), starting from day 11 after immunization (p.i.); EAE rats were injected intraperitoneally with the vehicle as a control group (Pihl-Jensen et al., 2021); and non-immunized rats i.p. injected with the vehicle were used as the naive control group (Kimura et al., 2017). From the date of immunization, changes in body weight, clinical signs, and neurological function scores were observed and recorded by two independent observers. The EAE model score uses the five-point scale: 0 = no clinical score; 1 = tail weakness; 2 = hind limb weakness; 3 = hind limb paralysis; 4 = forelimb paralysis; and 5 = moribund or death.

### Histopathological evaluation

The rats were sacrificed on day 17 p.i. After extensive perfusion with physiological saline solution, the optic nerves and retinas were removed and post-fixed with FAS eyeball fixative (Servicebio, Wuhan, China). The tissue was then embedded in paraffin and cut into 2–5 μm thick sections, dewaxed in xylene, and rehydrated. Hematoxylin–eosin (H&E) staining was used to detect inflammatory infiltration, and Luxol fast blue (LFB) staining was used to detect demyelination. The degree of infiltration of inflammatory cells and demyelination in the optic nerve was assessed by a double-blind investigator, similar to the open standard. For inflammation, 0 = no infiltration, 1 = a little cell infiltration of the optic nerve or optic nerve sheath, 2 = moderate infiltration, 3 = serious infiltration, and 4 = substantial infiltration. For demyelination, 0 = no demyelination, 1 = scattered demyelinating lesions, 2 = partial demyelinating lesion, and 3 = large number of demyelinating lesions. Scores of inflammation, infiltration, and demyelination were evaluated by Image-Pro Plus 6.0 software. Each experiment was repeated three times, and the results were averaged.

### Immunofluorescence double labeling

Briefly, non-specific binding was blocked with 3% bovine serum albumin (BSA) (Serotec, United Kingdom) and permeabilized with 0.3% Triton X-100 in 1% BSA-PBS for 30 min. The sections were then incubated in blocking solution at 4°C overnight with primary antibodies specific for rabbit anti-Nrf2 (1:100), rabbit anti-SIRT1 (1:100), rabbit anti-PGC-1α (1:300), and rabbit anti-TOMM20 (1:250) (all from Abcam, Cambridge, United Kingdom) and then incubated with secondary antibody donkey anti-rabbit FITC (1:200; IgG; Proteintech, Wuhan, China) at room temperature (RT) for 2 h. After being permeabilized with 1% BSA-PBS for 3 × 5 min, the sections were incubated with rabbit anti-Brn3a (1:100; IgG; Bioss, Beijing, China) specific primary antibody in blocking solution overnight at 4°C, then incubated with secondary antibody donkey anti-rabbit Cy3 (1:200; IgG; Proteintech) at RT for 2 h, mounted with 4’,6-diamidino-2-phenylindole (DAPI, 1:1,000; Roche, Basel, Switzerland), washed with PBS, cover-slipped, and examined under a fluorescence microscope (Leica Microsystem AG, Switzerland). As a negative control, additional sections were treated similarly, but the primary antibodies were omitted. All pictures were captured by a confocal microscope (Olympus Fluoview FV1000). For each group, ten sections were examined in a blinded fashion. Image-Pro Plus 6.0 software was used to calculate the quantification of target protein expression.

### Cell Culture of RGC-5

Rat retinal ganglion cells (RGC-5) were purchased from iCell Bioscience Inc., Shanghai, China. Cells were maintained in Dulbecco’s modified eagle medium (iCell Bioscience Inc., Shanghai) supplemented with 10% heat-inactivated fetal bovine serum (iCell Bioscience Inc., Shanghai) and in a humidified atmosphere containing 5% CO_2_ at 37°C. RGC-5 cells were seeded into 6-well plates. Cells were treated in four groups: for the control group, 2 ml complete medium was added. For the TNF-α group, 2 ml of TNF-α (MedChemExpress, Shanghai, China) at a concentration of 50 ng/ml was added to the medium. For the MAT group, 2 ml (50 ng/ml) of TNF-α and 100 μM of MAT were added to the medium. For the MAT + Ex-527 group, 2 ml (50 ng/ml) of TNF-α, 100 μM of MAT, and 38 nM of EX527 (Beyotime Biotechnology, Shanghai, China) were added. All groups were cultured for 48 h before harvest.

### Western Blot

After treatment, total protein from both cell lysate and the supernatant was isolated from N9 cells with RIPA lysis buffer (50 mM Tris-HCl, pH 7.4, 150 mM NaCl, 0.25% deoxycholic acid, 1% Nonidet P-40, one mM EDTA) including the protease and phosphatase inhibitor (Beyotime Biotechnology, Shanghai, China). For Western blot analysis, 8%–10% SDS-PAGE was used to resolve equal amounts of protein samples from both cell lysate and supernatant. The gel was transferred onto polyvinylidene fluoride (PVDF) membranes (Sigma-Aldrich, United States), and the membranes were blocked with 5% BSA in Tris-buffered saline containing 0.05% Tween-20 (TBST). Membranes were probed with primary antibodies at 4°C overnight. The following day, the incubated membranes were washed four times with TBST and then incubated with the horseradish peroxidase (HRP)-conjugated secondary antibodies. The antigen–antibody complex was screened by chemiluminescence using the Supersignal West Dura ECL reagent (Thermo Scientific, United States). Protein bands were detected with a densitometer (Bio-Rad, Shanghai). Band density analysis was performed with ImageJ software (National Institutes of Health, United States).

### Measurement of mitochondrial membrane potential (ΔΨM)

For the measurement of mitochondrial membrane potential (ΔΨM), cells were harvested and stained with JC-1 (Beyotime Biotechnology, Shanghai, China) and Rhodamine 123 (Beyotime Biotechnology, Shanghai, China) and were quantified by flow cytometry analysis as described previously (Pal et al., 2020). JC-1 dye is a lipophilic, cationic dye developed to detect ΔΨM in healthy and apoptotic cells. After two washes with phosphate-buffered saline (PBS) to remove media, trypsin-treated cells were harvested, washed again with PBS, and then incubated in 500 μL JC-1 dye for 20 min. Samples were then washed with PBS once and analyzed immediately by an FACS analyzer (Luminex, Guava^®^ easyCyteTM). Rhodamine 123 is a cationic fluorescent dye. Treated cells were harvested and washed once with PBS and incubated with 500 μl of Rho123 dye (2 μM) for 30 min at a 37°C shaker incubator. After incubation, cells were washed with PBS and analyzed immediately by an FACS analyzer.

### Statistical analyses

Multiple comparisons were performed using one-way ANOVA, followed by the Student–Newman–Keuls test. Clinical EAE scores and mean body weight were compared at individual time points between vehicle- and MAT-treated rats. Statistical software (GraphPad Prism 7.0, IBM SPSS Statistics 27) was used for statistical analyses; *p* < 0.05 was considered significant. Given that ON can occur bilaterally or unilaterally in either eye (Khan et al., 2017), each eye was used as an independent data point for all histological experiments.

## Results

### MAT alleviated the severity of optic neuropathology

As we reported before, MAT effectively suppresses CNS inflammation, demyelination, and axonal loss in optic nerves, as well as RGC apoptosis in EON (Kang et al., 2021). Briefly, we first induced an animal model of EON in Wister rats and then treated it from day 11 p.i., which resulted in a significant reduction in clinical scores for EAE and mitigation in weight loss in the MAT-treated group compared to saline-treated model rats. In order to verify the correlation between the clinical signs of EAE and EON, optic nerve transverses of all groups were examined by HE and LFB staining. Inflammatory infiltrates were diffusely distributed in the optic nerve of vehicle-treated rats, and this infiltration was significantly inhibited by MAT treatment. Furthermore, MAT treatment significantly reduced optic nerve demyelination compared to the EAE group (data shown in Supplementary Material).

### MAT promoted SIRT1 expression in RGCs

**FIGURE 1:**
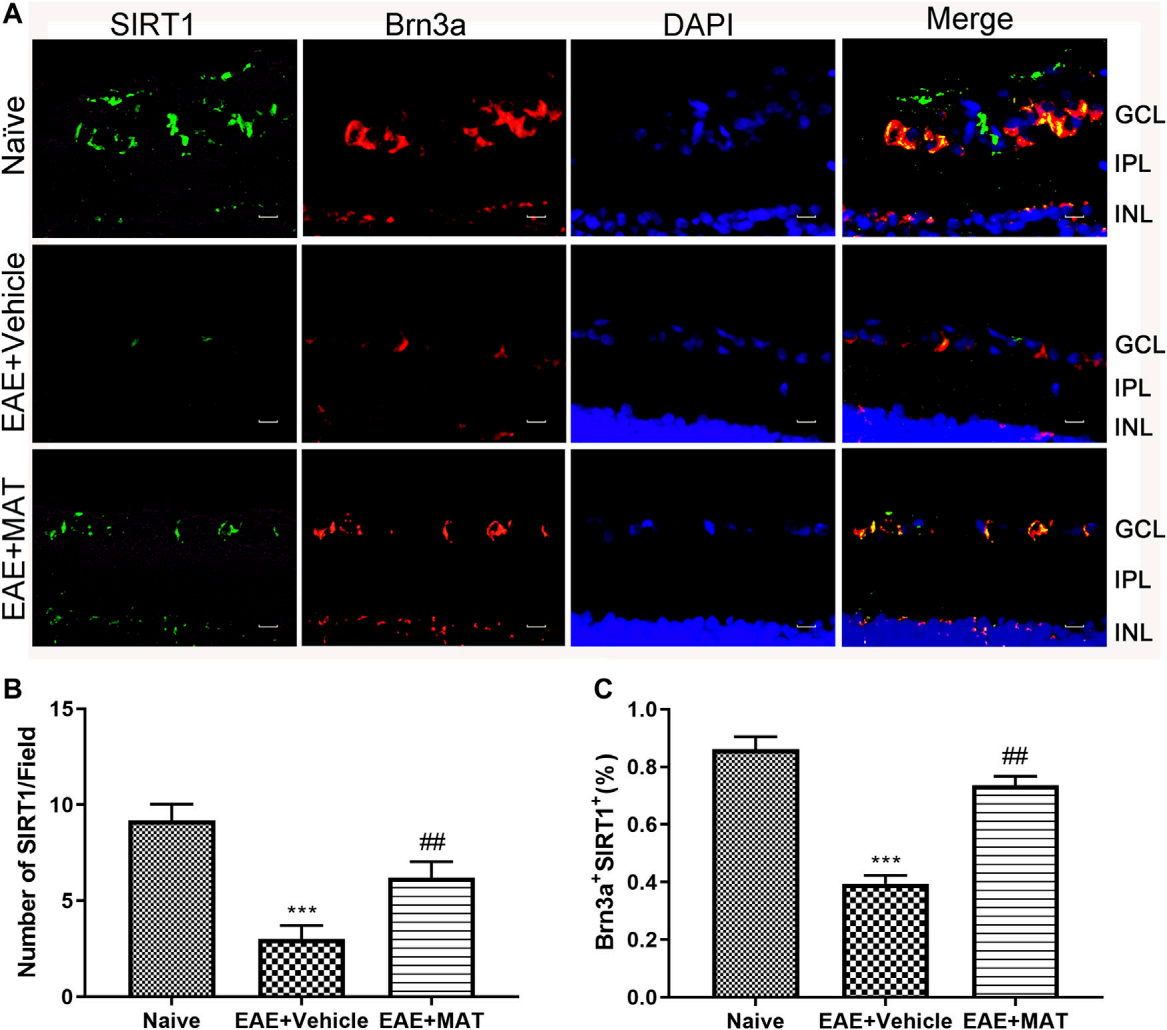
MAT promoted SIRT1 expression in RGCs. Eyeballs were harvested from naive rats, MAT- and vehicle-treated EAE rats. **(A)** Immunofluorescence double staining suggested that SIRT1 (FITC, green) were colocalized with Brn3a (Cy3, red) in the eyeball retina of EAE rats (200×). Scale bars, 10 μm. **(B)** Quantitative analysis of the number of positive cells. **(C)** Quantitative analysis of the rate of positive cells. Data represent mean ± SD; n = 10 rats per group. ** *p* < 0.01, *** *p* < 0.001, comparison between naive and vehicle-treated EAE groups. ## *p* < 0.01, comparison between vehicle- and MAT-treated EAE groups.

To investigate the effect of MAT treatment on SIRT1 expression, we tested the number of cells that colocalized SIRT1 with Brn3a (RGCs marker) by immunofluorescence double staining. As shown in Figure 1A, it was observed on the paraffin section of the sagittal plane of the eye that the expression of SIRT1 was reduced in the vehicle-treated EAE rats compared with the naive rats. In contrast, its expression was significantly increased after treatment with MAT (Figures 1B,C). These results indicate an upregulating effect of MAT on the SIRT1 expression in RGCs.

### Increased expression of Nrf2 and PGC-1α by MAT treatment

**FIGURE 2:**
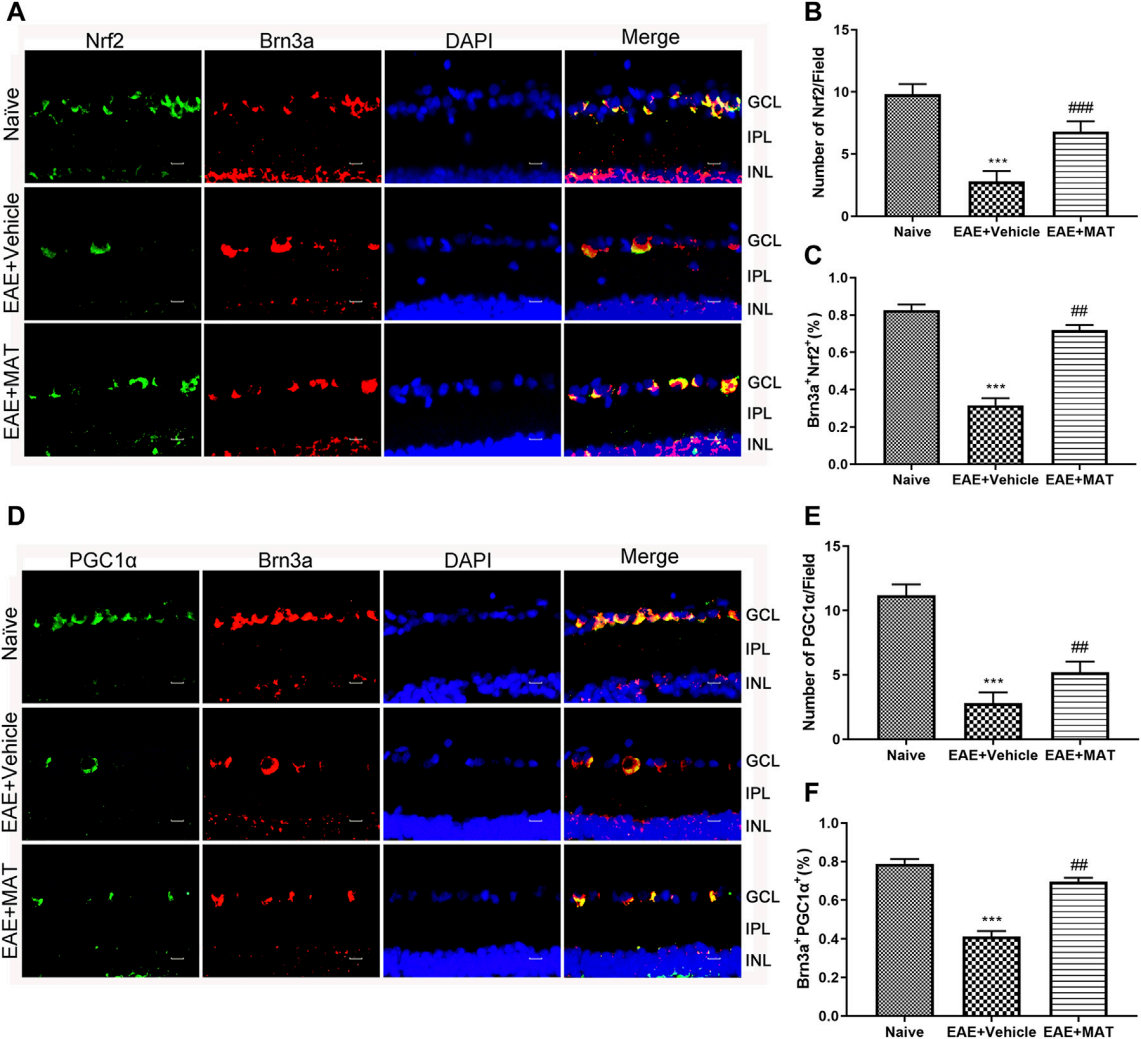
MAT elevated the expression of Nrf2 and PGC-1α. Eyeballs were harvested from naive rats, MAT- and vehicle-treated rats. **(A)** Immunofluorescence double staining suggested that Nrf2 (FITC, green) were colocalized with Brn3a (Cy3, red) (200×). Scale bars, 10 μm. **(B)** Quantitative analysis of the number of positive cells. **(C)** Quantitative analysis of the rate of positive cells. **(D)** Immunofluorescence double staining suggested that PGC-1α (FITC, green) were colocalized with Brn3a (Cy3, red) in the eyeball retina (200×). Scale bars, 10 μm. **(E)** Quantitative analysis of the number of positive cells. **(F)** Quantitative analysis of the rate of positive cells. Symbols represent mean ± SD; n = 10 rats per group. **p < 0.01, ***p < 0.001, comparison between naive and vehicle-treated groups. ##*p* < 0.01, comparison between vehicle- and MAT-treated EAE groups.

Nrf2 is a key factor in the oxidative defense system, and SIRT1 is involved in the expression and activation of Nrf2. We, therefore, studied Nrf2 expression on the retina by immunofluorescence double staining to illustrate the effect of MAT on oxidative stress in ON (Figure 2A). The expression level of Nrf2 in the vehicle group was reduced compared to that in the naive group. This expression was significantly increased in the MAT-treated group compared to that in the vehicle-treated group (Figures 2B,C).

The PGC-1α expression has been considered to play a non-negligible role in mitochondrial biosynthesis. Therefore, we used immunofluorescence double staining to study the PGC-1α in the retina on the ON model (Figure 2D). Our results showed that the expression of PGC-1α in the immunized groups was significantly lower than in the normal group, whereas its expression in the MAT treatment group was markedly higher than in the vehicle-treated group (Figures 2E,F). These results indicate that MAT can promote the expression of PGC-1α.

### MAT could promote Nrf2/PCG-1α expression in RGC-5 by upregulating the SIRT1 expression

**FIGURE 3:**
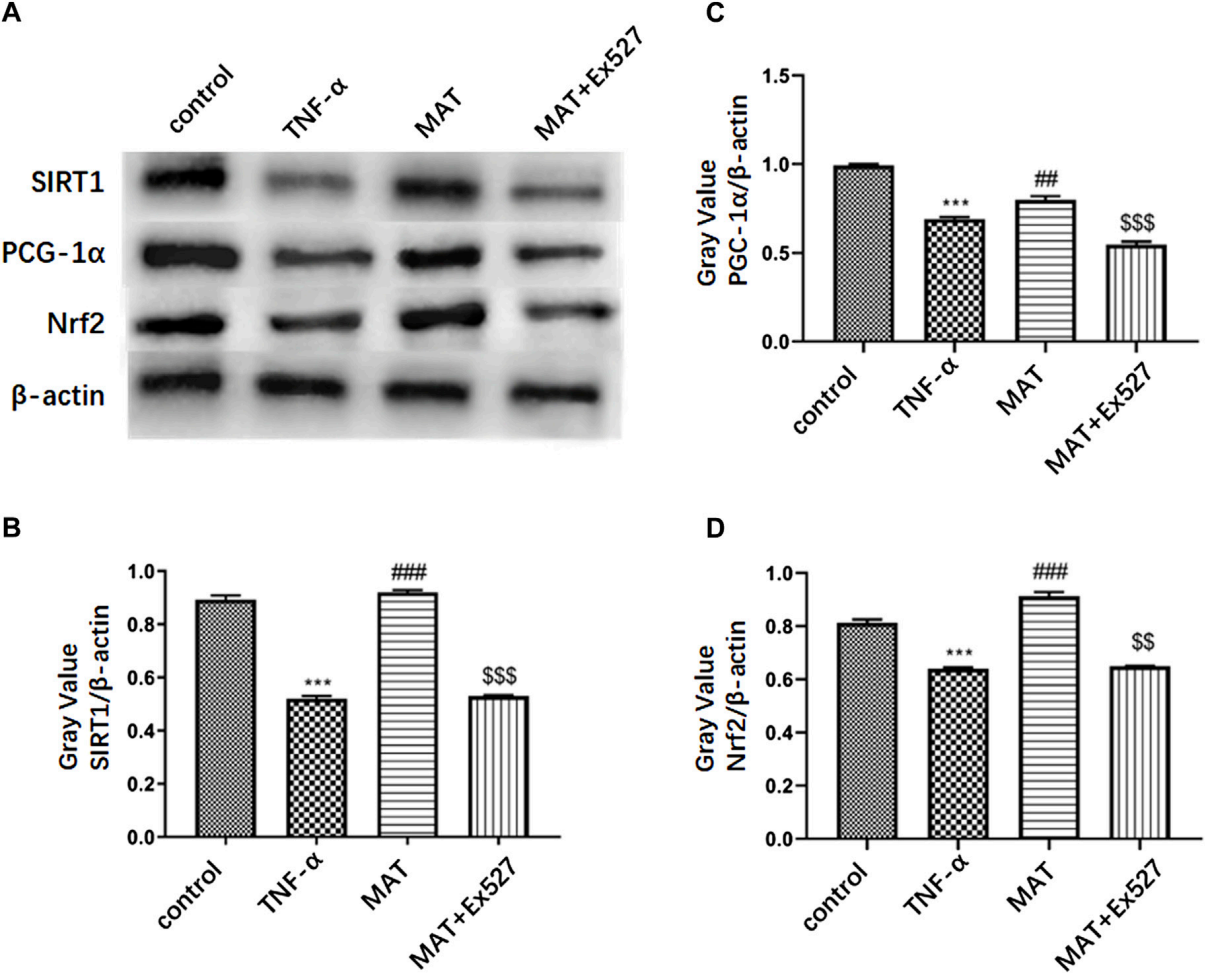
MAT could promote Nrf2/ PCG-1α expression in RGC-5 through upregulating SIRT1 expression. **(A)** PCG-1α and Nrf2 protein expression in RGC cells treated by TNF-α were attenuated compared to control group, as well as SIRT1 expression. MAT co-treatment could improve the expression of SIRT1, PCG-1α and Nrf2 significantly. This effect could be revised by EX527, an SIRT1 inhibitor. **(B-D)** Quantitative analysis of the relative expression of each molecule. Symbols represent mean ± SD; ** *p* < 0.01, ***p < 0.001, comparison between control and TNF-α groups. ##*p* < 0.01, ### *p* < 0.001, comparison between TNF-α and MAT groups. $$*p* < 0.01, $$$ *p* < 0.001 comparison between MAT and MAT+EX527 groups.

To confirm that MAT could prevent RGC death through the SIRT1-PCG-1α/Nrf2 pathway, we used the RGC-5 cell line to perform the experiment. The PCG-1α and Nrf2 protein expression in RGC treated by TNF-α were attenuated compared to the control group, as well as the SIRT1 expression. MAT co-treatment could improve the expression of SIRT1, PCG-1α, and Nrf2 significantly. This effect could be revised by EX527, a SIRT1 inhibitor (Figure 3).

### MAT affected mitochondrial biosynthesis

**FIGURE 4:**
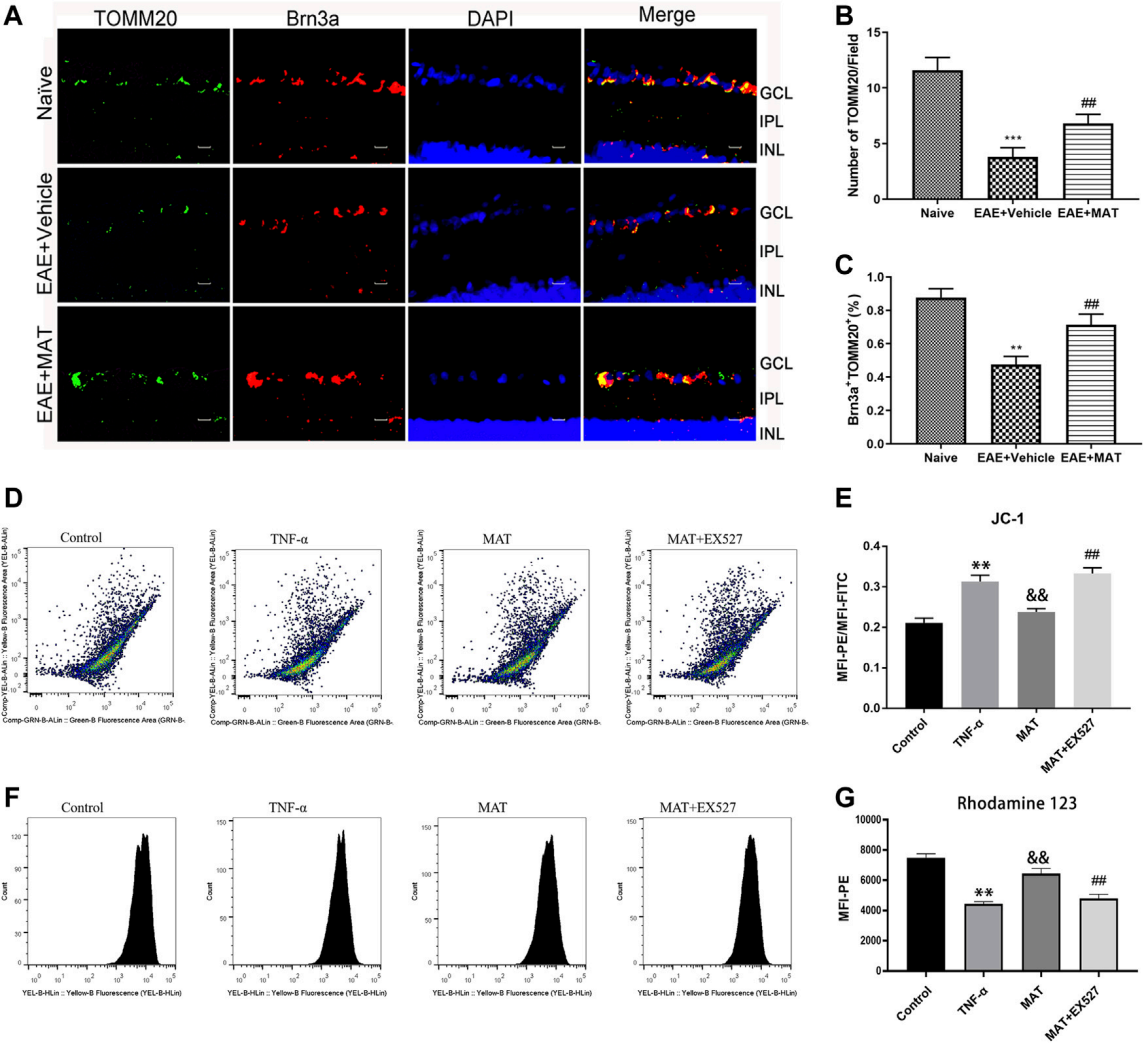
MAT motivated mitochondrial biosynthesis. Eyeballs were harvested from naive rats, MAT- and vehicle-treated EAE rats. **(A)** Immunofluorescence double staining suggested that TOMM20 (FITC, green) were colocalized with Brn3a (Cy3, red) in the eyeball retina of EAE rats. Scale bars, 10 μm. **(B)** Quantitative analysis of the number of positive cells. **(C)** Quantitative analysis of the rate of positive cells. Symbols represent mean ± SD; n = 10 rats per group. **p < 0.01, ***p < 0.001, comparison between naive and vehicle-treated EAE groups. ##*p* < 0.01, comparison between vehicle- and MAT-treated EAE groups. **(D-G)** TNF-α expose increase in loss of mitochondrial membrane potential (ΔѰM) in retinal cells. FACS analysis using JC-1 dye and Rhodamine-123 dye showing increase in mitochondrial membrane potential ((ΔѰM) with MAT treatmen in RGC-5 cells and this effect could be attenuated by EX527. Symbols represent mean ± SD; ** *p* < 0.01, comparison between control and TNF-α groups. &&*p* < 0.01, comparison between TNF-α and MAT groups. ##*p* < 0.01 comparison between MAT and MAT+EX527 groups.

Previous studies had shown that MAT protected mitochondrial membrane integrity by inhibiting cytochrome c (Cyt c) release (Wang et al., 2019). Therefore, we decided to explore the effects of MAT on mitochondria in the retina. We determined the expression of TOMM20, a mitochondrial outer membrane transporter commonly used to label mitochondria, in RGCs by immunofluorescence double staining (Figure 4A). The percentage of TOMM20+ Brn3a+ cells in the retina was remarkably reduced during the disease period. However, it was increased in the MAT treatment group (Figures 4B,C). Thus, MAT can enhance mitochondrial biosynthesis in the retina.

Mitochondrial membrane potential (ΔѰM) reflects the functional status of the mitochondrion. We used JC-1 and Rho123 for the qualitative measurement of ΔѰM in RGC-5 cells. Cells with healthy mitochondria show high ΔѰM, whereas cells with damaged mitochondria show low ΔѰM. FACS analysis showed that, with the exposure of TNF-α to RGC-5 cells for 48 h, a significant decrease in ΔѰM was observed, and this was revised by 100 μM MAT, whose effect could be attenuated by Ex527 (Figures 4D,E). To support these findings, we checked the ΔѰM level using Rho123 dye. There was a gradual decrease in the ΔѰM level in the TNF-α group. However, the decreased level of mitochondrial ΔѰM upon TNF-α exposure was revised after the treatment of MAT, and this effect could be attenuated by the SIRT1 inhibitor (Figures 4F,G).

## Discussion

Axons of RGCs, which form the optic nerve, are demyelinated in various optic neuropathies, including ON (Wang et al., 2018), and have very limited spontaneous regeneration after injury (Benowitz et al., 2017). ON, which usually occurs in patients with MS and its animal model, EAE (Quinn et al., 2011; Murphy et al., 2020), can cause optic nerve damage and subsequently the death of RGCs (Moore and Goldberg, 2010). It has been recently shown that MAT can limit the inflammation and demyelination of the optic nerve and reduce RGCs apoptosis (Kang et al., 2021). However, the mechanism of this natural alkaloid during this process is still unclear. Herein, we show that the MAT treatment induces SIRT1, PGC-1α, and Nrf2 expression; promotes mitochondrial biosynthesis; and reduces oxidative stress, thereby preventing the loss of RGCs and exerting protective effects on the optic nerve in ON.

Mitochondria are cytoplasmic organelles responsible for producing adenosine triphosphate (ATP), and they play an important role in regulating cellular calcium metabolism, reactive oxygen species (ROS) production, and apoptosis (Mancini et al., 2018). It has been discussed that during the progression of optic neuritis, the oxidative injury to the mitochondrion began prior to inflammatory cell infiltration and continued (Guy, 2008). When the mitochondrial respiratory chain synthesizes ATP, it also produces ROS, mainly in the shape of superoxide, because of the leakage of electrons to oxygen molecules. The increase in mitochondrial electron transport chain activity usually increases the production of by-products ATP and ROS, while the amassing of superoxide in the mitochondria is noxious to cells (Packialakshmi and Zhou, 2018). Due to the central metabolic function of mitochondria and its involvement in the pathophysiology of neurodegenerative diseases, diabetes, and cancer, it has become the focus of basic and translational research (Kappler et al., 2019). Mitochondria are reported to be abundant in the RGCs, and the number of mitochondria in unmyelinated axons is higher than in myelinated axons (Kitaoka et al., 2013). Talla et al. also showed that the reduced activity of the optic nerve complex I in EAE mice increases mitochondrial oxidative stress, which leads to neurodegeneration related to permanent vision loss (Talla et al., 2015). Among molecules that regulate mitochondria function, the SIRT1 expression could prevent the loss of RGC by reducing oxidative stress and maintaining energy homeostasis (Munemasa and Kitaoka, 2015). Most capabilities of SIRT1 happen in the nucleus, where SIRT1 deacetylates histones or other proteins, such as transcription factors or chromatin remodeling proteins (Kim et al., 2015). Although SIRT1 is mostly located in the nucleus, the regulation of mitochondrial biogenesis and function primarily depends on the distribution of SIRT1 in the cytoplasm and mitochondria (Aquilano et al., 2013). Furthermore, SIRT1 regulates cell survival and apoptosis by mediating the deacetylation of p53 (Kulkarni et al., 2014; Yarahmadi et al., 2019), a tumor suppressor gene that contributes to the oligomerization of pro-apoptotic proteins in mitochondria through transcription-dependent and non-transcription-dependent pathways, which may induce mitochondrial outer membrane permeability and mitochondrial Cyt c release (Tu et al., 2018; Vidhyapriya et al., 2018). Our experimental results show significantly enhanced expression of SIRT1 in RGCs after MAT treatment both *in vivo* and *in vitro*, suggesting that MAT may regulate mitochondrial function by upregulating the SIRT1 expression of RGCs, thereby preventing their loss in ON.

Mitochondrial oxidative stress is generally considered to be the major agent of many neurodegenerative diseases such as MS and ON (Khan et al., 2017). Nrf2 is an important transcription factor of the mitochondrial endogenous antioxidant pathway, which affects the expression of multiple antioxidant pathways, including glutathione and cytoprotective genes (Osborne et al., 2016). Under redox-equilibrium cellular conditions, Nrf2 is sequestered in the cytoplasm and undergoes proteasome-mediated degradation (McMahon et al., 2003). During oxidative stress, modification of key binding proteins allows Nrf2 to dissociate and enter the nucleus, recruiting transcription mechanisms to participate in the antioxidant response elements (AREs) and stimulating transcription of target genes associated with antioxidant defense and cellular detoxification (Chen et al., 2015). In addition, the activation of the Nrf2 antioxidant pathway and nuclear accumulation are also regulated by SIRT1 (Zhao et al., 2019). Nrf2 is referred to as the molecular switch of Nrf2/Keap1/ARE signaling and is also a paramount part of the ROS signaling pathway, which can be activated by oxidative stress inducers (Liu et al., 2018). The activation of the Nrf2/ARE signaling pathway is also a self-defense and protection mechanism of cells in response to oxidative stress (Cheng et al., 2017; Yu et al., 2019) and has a neuroprotective effect in EAE-related ON (Larabee et al., 2016). To explore the effect of MAT on Nrf2 expression in RGCs of EAE rats, we found that the expression of Nrf2 was significantly increased upon MAT treatment, suggesting that MAT promotes mitochondrial biosynthesis and reduces oxidative stress, which may be related to the promotion of Nrf2 expression. Moreover, the Nrf2 expression was inhibited by EX527, a SIRT1 inhibitor, suggesting MAT upregulated the Nrf2 expression in RGCs by activating the SIRT1 pathway.

It has been shown that the activity of Nrf1 and Nrf2 can be induced by the PGC-1 transcriptional coactivators family, composed of PGC-1α, PGC-1β, and PGC-1-related coactivators. This activity leads to the transactivation of many genes encoding mitochondrial biogenic specific proteins (Selvakumar et al., 2018). PGC-1α is a major regulator of cellular metabolism and is involved in guiding the expression of nuclear regulatory genes related to mitochondrial biogenesis and antioxidant stress (St-Pierre et al., 2006). PGC-1α has no DNA binding activity of its own but can jointly activate a large number of transcription factors; for example, its interaction with NRFs can promote mitochondrial gene expression and proliferation (Bruns et al., 2019). Consequently, PGC-1α is an intermediary of oxidative phosphorylation and mitochondrial biogenesis and is generally considered the main regulator of mitochondrial function in mammals (LeBleu et al., 2014). The deacetylation state of PGC-1α is the activation state during mitochondrial biogenesis (Price et al., 2012). SIRT1 directly interacts with PGC-1α to regulate PGC-1α activity, and with the increase in the transcriptional activity of PGC-1α, mitochondrial gene transcription and mitochondrial biogenesis are also enhanced (Ma et al., 2017). By co-activating with Nrf2, PGC-1α modulates the expression of metabolic genes in the nuclear and mitochondrial genomes and thus serves as a crucial regulator of accommodative response to oxidative stress (Sharma et al., 2015). PGC-1α is also an important regulator of the mitochondrial endogenous antioxidant defense system, which works by regulating numerous antioxidant proteins, independent of Nrf2 (Osborne et al., 2016). Reduced PGC-1α expression in the retina is involved in all major processes of retinal damage and subsequent repair (Egger et al., 2012). Moreover, the PGC-1α signaling pathway is a significant regulator of astrocyte reactivity and RGC homeostasis as it adjusts the pathogenic sensitivity of the inner retina to metabolic and oxidative damage (Guo et al., 2014). Consistent with these observations, PGC-1α expression in the retina of ON rats and RGC-5 treated with TNF-α both were significantly increased after MAT treatment and could be attenuated by EX527, suggesting that MAT may promote mitochondrial biogenesis and maintain RGC homeostasis by SIRT1/PGC-1α pathway.

In conclusion, our study shows that MAT can effectively inhibit the disease progression of ON and protect RGCs. MAT activates the SIRT1, PGC-1α, and Nrf2 expression in the retina to promote mitochondrial biosynthesis and reduce oxidative stress, which could be revised by the SIRT1 inhibitor. Thus, we believe that MAT treatment could protect RGCs from apoptosis in ON by the SIRT1-PGC-1α/Nrf2 pathway.

## Data Availability

The raw data supporting the conclusions of this article will be made available by the authors without undue reservation.
