# Supplementary material for: Matrine promotes mitochondrial biosynthesis and reduces oxidative stress in experimental optic neuritis
Source: Front Pharmacol. 2022 Sep 27;13:936632. doi: 10.3389/fphar.2022.936632 (PMC9552203; doi:10.3389/fphar.2022.936632)
Supplement: Supplementary file 1 [file Image1.pdf]

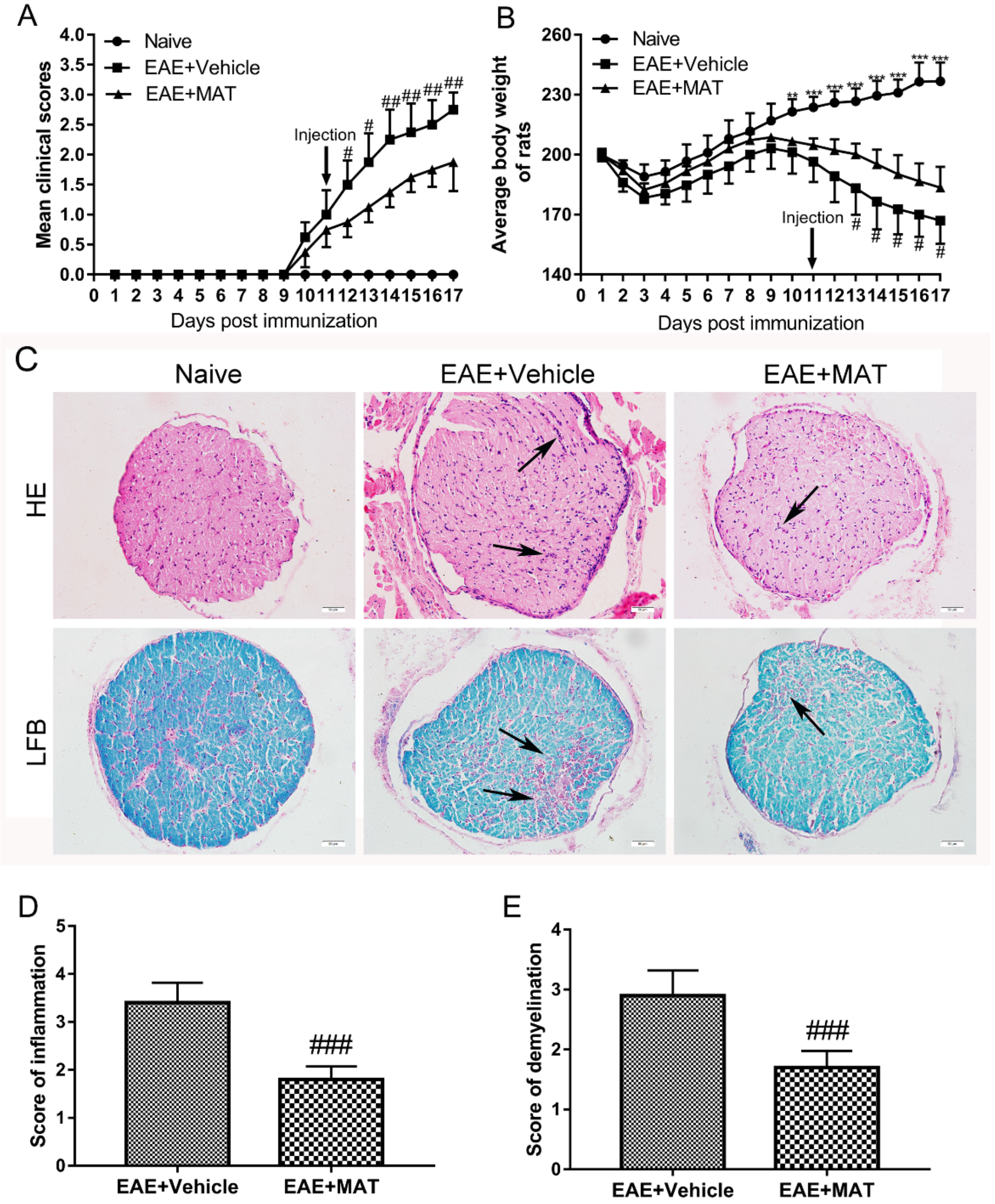


**Figure 1.** MAT improved clinical presentation and optic neuropathology of EAE. Wistar rats were immunized and clinical score was assessed as described in the Materials and Methods section. The effects of MAT on clinical score (A) and mean body weight (B) were examined daily. (C) Rats were sacrificed on day 17 p.i. The optic nerve and eyeballs were collected after extensive perfusion for H&E staining (100×， for inflammation) and LFB staining (100×， for demyelination). Scales bars, 50μm. Results were analyzed statistically for inflammation (D) and demyelination (E). Data represent mean ± SD (n=10 rats per group). $**P < 0.01$, $***P < 0.001$, comparison between naive and vehicle-treated EAE groups. $\#P < 0.05$, $\#\#P < 0.01$, $\#\#\#P < 0.001$, comparison between vehicle- and MAT-treated EAE groups.
